# Expression profile of the entire family of *Adhesion *G protein-coupled receptors in mouse and rat

**DOI:** 10.1186/1471-2202-9-43

**Published:** 2008-04-29

**Authors:** Tatjana Haitina, Fredrik Olsson, Olga Stephansson, Johan Alsiö, Erika Roman, Ted Ebendal, Helgi B Schiöth, Robert Fredriksson

**Affiliations:** 1 Department of Neuroscience, Unit of Functional Pharmacology, Uppsala University, BMC, 75124 Uppsala, Sweden; 2 Department of Pharmaceutical Biosciences, Division of Pharmacology, Uppsala University, BMC, 75124 Uppsala, Sweden; 3 Department of Neuroscience, Unit of Developmental Neuroscience, Uppsala University, BMC, 75124 Uppsala, Sweden

## Abstract

**Background:**

The *Adhesion *G protein-coupled receptors (GPCRs) are membrane-bound receptors with long N termini. This family has 33 members in humans. Several *Adhesion *GPCRs are known to have important physiological functions in CNS development and immune system response mediated by large cell surface ligands. However, the majority of *Adhesion *GPCRs are still poorly studied orphans with unknown functions.

**Results:**

In this study we performed the extensive tissue localization analysis of the entire *Adhesion *GPCR family in rat and mouse. By applying the quantitative real-time PCR technique we have produced comparable expression profile for each of the members in the *Adhesion *family. The results are compared with literature data and data from the Allen Brain Atlas project. Our results suggest that the majority of the *Adhesion *GPCRs are either expressed in the CNS or ubiquitously. In addition the *Adhesion *GPCRs from the same phylogenetic group have either predominant CNS or peripheral expression, although each of their expression profile is unique.

**Conclusion:**

Our findings indicate that many of *Adhesion *GPCRs are expressed, and most probably, have function in CNS. The related *Adhesion *GPCRs are well conserved in their structure and interestingly have considerable overlap in their expression profiles, suggesting similarities among the physiological roles for members within many of the phylogenetically related clusters.

## Background

G protein-coupled receptors (GPCRs) can be divided into five subfamilies according to the GRAFS classification system: *Glutamate, Rhodopsin, Adhesion, Frizzled/Taste2 *and *Secretin *[1]. The *Adhesion *GPCRs, also known as long N-terminal seven transmembrane receptors related to family B (LNB-TM7) and epidermal growth factor seven transmembrane (EGF-TM7) receptors. This family is the second largest GPCR family with 33 members in humans and 30 members in mice and rats. The endogenous ligands are discovered for BAI1, GPR56, CD97 and EMR2, while the rest of the Adhesion GPCRs remain orphans.

The *Adhesion *receptors are characterized by long N-termini with complex domain architecture including GPCR proteolytic site (GPS), epidermal growth factor, thrombospondin, pentraxin, immunoglobulin, olfactomedin, cadherin domains [2]. Several of the domains in the N-termini have been shown to have cell adhesion functions and to be responsible for cell-to-cell and cell-to-matrix interactions [3,4]. The GPS domain is thought to be an intracellular cleavage motif, which is crucial for transport of the receptor from ER to the membrane [5]. The Adhesion GPCRs are cleaved into two parts: N-terminal and 7TM part with the C-terminal. Nevertheless it has been reported that the N-terminal is responsible for ligand binding and is able to re-associate with the rest of the receptor and in this way initiate intracellular signalling cascade [6]. The genes coding for *Adhesion *GPCRs are difficult to study due to their complex genomic structure and a large number of exons.

**Figure 1:**
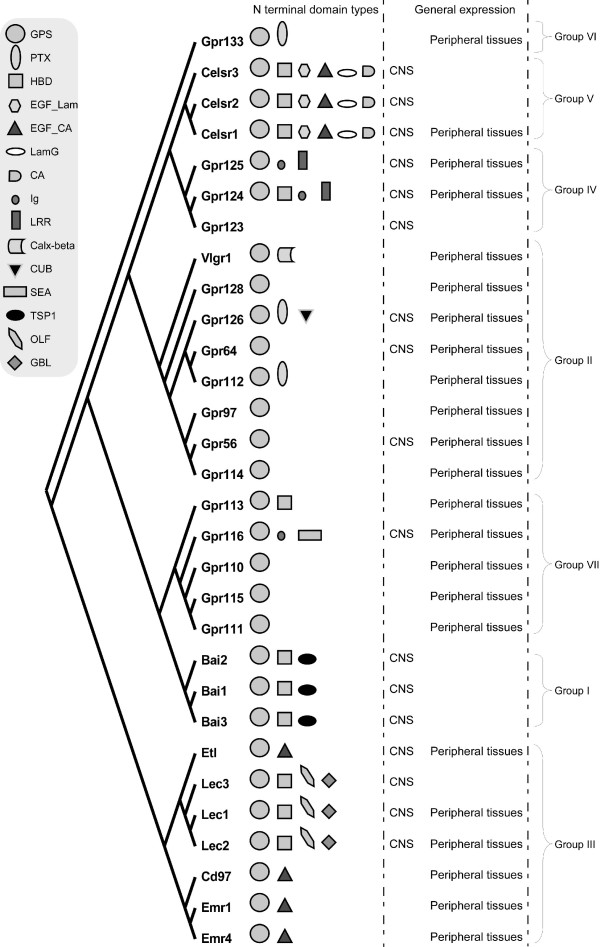
**A phylogenetic tree, N terminal domains and general expression of *Adhesion *GPCRs**. A consensus phylogenetic tree of 7TM regions of mouse *Adhesion *GPCRs was generated by Maximum – Likelihood analysis (Phylip 3.67). The N-terminal domains of *Adhesion *GPCRs are as follows: GPS (GPCR proteolytic site), PTX (Pentraxin domain), HBD (hormone binding domain), EGF_Lam (laminin type epidermal growth factor domain), EGF_CA (EGF, calcium binding domain), LamG (laminin G domain), CA (cadherin repeats), Ig (immunoglobulin domain), LRR(leucine rich repeat), Calx-beta domain, CUB (C1r/C1 s urinary EGF and bone morphogenetic domain), SEA (sea urchin sperm protein domain), TSP1 (thrombospondin repeats, type 1), OLF (olfactomedin domain) and GBL (galactose binding lectin domain). The displayed predominant expression is based on obtained real-time PCR data.

The *Adhesion *GPCRs can be divided into seven groups according to phylogenetic analysis (Figure 1) [2]. The brain angiogenesis inhibitors (BAIs) 1–3 form group I and are known to be mainly expressed in the brain. The N-terminal of BAI receptors contains GPS, hormone binding domain and thrombospondin repeats which are likely to be involved in the angiogenesis inhibitor function of BAIs. The role of BAIs in tumor suppression has been closely investigated as angiogenesis is an essential part of tumor formation [7]. BAI1 has been recently reported as a receptor for phospatidylserine involved in recognition and internalization of apoptotic cells [8]. GPR56 (group II) is another *Adhesion *GPCR with a role in tumor development [9]. Mutations in GPR56 have also been reported to cause human brain malformations [10]. It is described that GPR56 is able to build complex with cell-surface protein tetraspanin CD81 and Gα_q/11 _and is able to bind tissue transglutaminase, TG2 [11,12].

GPR64 also called HE6 (group II), unlike GPR56, has its only known function in the periphery and disruption of this gene results in mouse male infertility [13]. The very large G-protein-coupled receptor (VLGR1) is important for normal development of auditory hair bundles in the inner ear [14]. The functions of other five members of group II are not known.

The *Adhesion *receptors forming group III can be divided into two groups according to the architecture of their N-termini. EMR1, EMR2, EMR3, EMR4, CD97 and ETL receptors have all a variable number of epidermal growth factor (EGF) and calcium binding domains and are reported to be important components of the immune system [2]. EMR2 and EMR3 are missing in the mouse and rat genomes. CD55 and chondroitin sulphate have been described as ligands for CD97, while chondroitin sulphate is also a ligand for EMR2. It is also suggested that EGF domains in the N-termini of CD97 are essential for CD55 binding. [15,16]. LEC1, LEC2 and LEC3 on the other hand are proposed to play more central roles like synaptic cell adhesion [17] and have a different repertoire of domains in the N-termini (hormone binding, olfactomedin and galactose binding lectin domains). LEC2 has been reported to bind α-latrotoxin from black widow spider venom [5].

GPR123, GPR124 and GPR125 belong to group IV of *Adhesion *GPCRs. GPR123 has been reported to have specific expression in the brain [18]. GPR125 was recently presented as a stem cell marker with a possible therapeutic use [19]. Both these receptors have protein-protein interaction domains in the form of leucine rich repeats, while GPR124 and GPR125 have immunoglobulin domains in N-termini as well.

CELSR1–3 receptors (group V) have the most complex domain structure with cadherin repeats, two types of EGF domains, laminin G and hormone binding domains. CELSR receptors are thought to have crucial functions in CNS development. Mutations in CELSR1 cause abnormal neural tube development in mice [20]. CELSR2 and CELSR3 seem to have opposite effects in neurite growth regulation; CELSR2 is enhancing neurite growth while CELSR3 is suppressing it. [21]. Unlike CELSR receptors, the members of group VI, GPR133 and GPR144 as well as group VII, GPR110, GPR111, GPR113, GPR115 and GPR116 do not have any discovered function.

The majority of *Adhesion *GPCRs are poorly studied, with specific expression patterns and physiological functions yet to be discovered. Until today, the published expression data on many *Adhesion *GPCR members is limited to a few tissues and is performed by different methods with varying sensitivity. Therefore, an overall expression analysis of *Adhesion *GPCRs is useful for comparison of expression and identifying specific patterns. It is also an important step towards finding the functions of the poorly studied receptors.

In this study we report an extensive tissue localization analysis of the entire *Adhesion *GPCR family in rat and mouse. By applying the quantitative real-time PCR technique we have produced comparable expression patterns for each *Adhesion *family member. We have compared expression patterns between rat and mouse and recent *in situ *hybridization data for mouse from the large Allen Brain Atlas project [22] as well as human and mouse ESTs [23]. We describe similarities in *Adhesion *GPCR expression patterns between rat and mouse as well as within groups of *Adhesion *GPCRs.

## Results

The mouse *Adhesion *GPCR gene sequences were downloaded from GenBank (Additional File 2) and 7TM regions were identified with Conserved Domain Database [24]. The tree calculations were carried out using ClustalW alignment of TM regions, which was bootstrapped 100 times and distances were calculated with PROML from Phylip 3.67 package. The Maximum-Likelihood consensus tree is presented in Figure 1.

**Figure 2:**
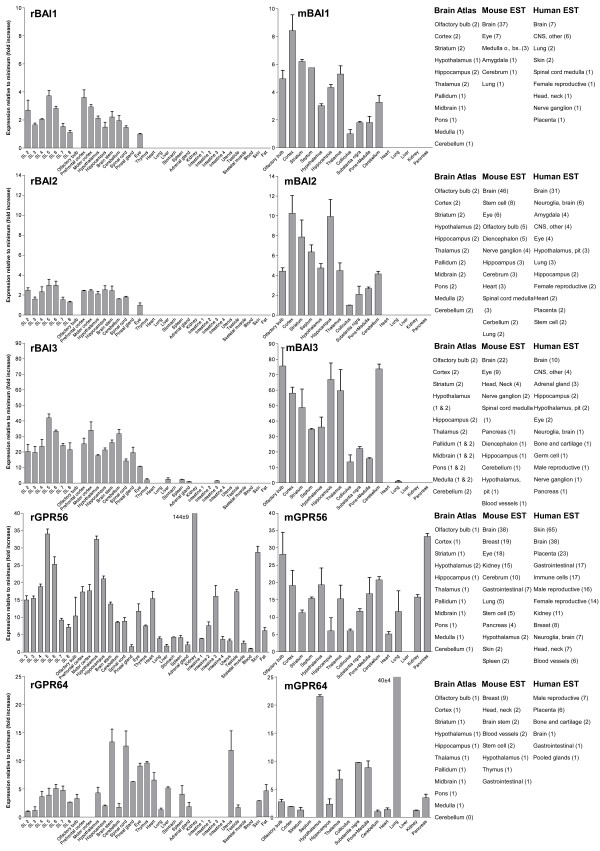
**Expression data of *Adhesion *GPCRs**. Relative expression of rat (r) and mouse (m) *Adhesion *GPCRs: BAI1, BAI2, BAI3, GPR56 and GPR64 obtained with quantitative real-time PCR. Error bars display standard deviation. The coronal sections of rat brain, marked SL2–SL8, are displayed in Additional File 1. For comparison, in situ hybridization data from mouse (0-no expression, 1-low expression and 2-high expression) [22] as well as mouse and human EST data [23] are displayed on the right side of the figure.

**Figure 3:**
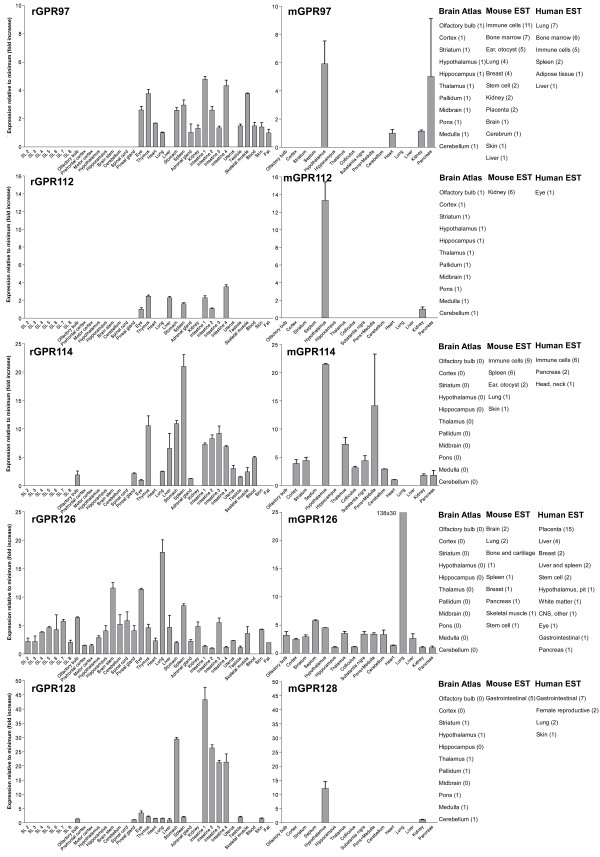
**Expression data of *Adhesion *GPCRs**. Relative expression of rat (r) and mouse (m) *Adhesion *GPCRs: GPR97, GPR112, GPR114, GPR126 and GPR128 obtained with quantitative real-time PCR. Error bars display standard deviation. The coronal sections of rat brain, marked SL2–SL8, are displayed in Additional File 1. For comparison, in situ hybridization data from mouse (0-no expression, 1-low expression and 2-high expression) [22] as well as mouse and human EST data [23] are displayed on the right side of the figure.

**Figure 4:**
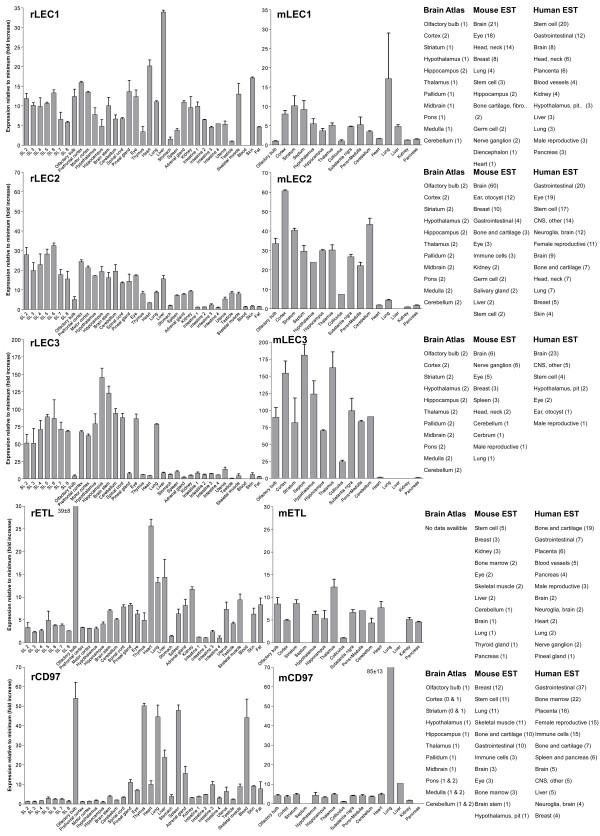
**Expression data of *Adhesion *GPCRs**. Relative expression of rat (r) and mouse (m) *Adhesion *GPCRs: LEC1, LEC2, LEC3, ETL and CD97 obtained with quantitative real-time PCR. Error bars display standard deviation. The coronal sections of rat brain, marked SL2–SL8, are displayed in Additional File 1. For comparison, in situ hybridization data from mouse (0-no expression, 1-low expression and 2-high expression) [22] as well as mouse and human EST data [23] are displayed on the right side of the figure.

**Figure 5:**
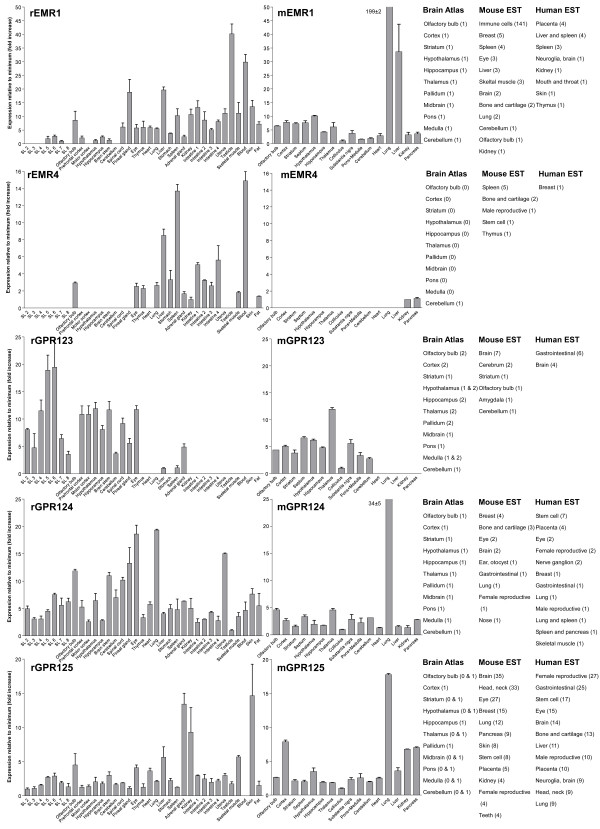
**Expression data of *Adhesion *GPCRs**. Relative expression of rat (r) and mouse (m) *Adhesion *GPCRs: EMR1, EMR4, GPR123, GPR124 and GPR125 obtained with quantitative real-time PCR. Error bars display standard deviation. The coronal sections of rat brain, marked SL2–SL8, are displayed in Additional File 1. For comparison, in situ hybridization data from mouse (0-no expression, 1-low expression and 2-high expression) [22] as well as mouse and human EST data [23] are displayed on the right side of the figure.

**Figure 6:**
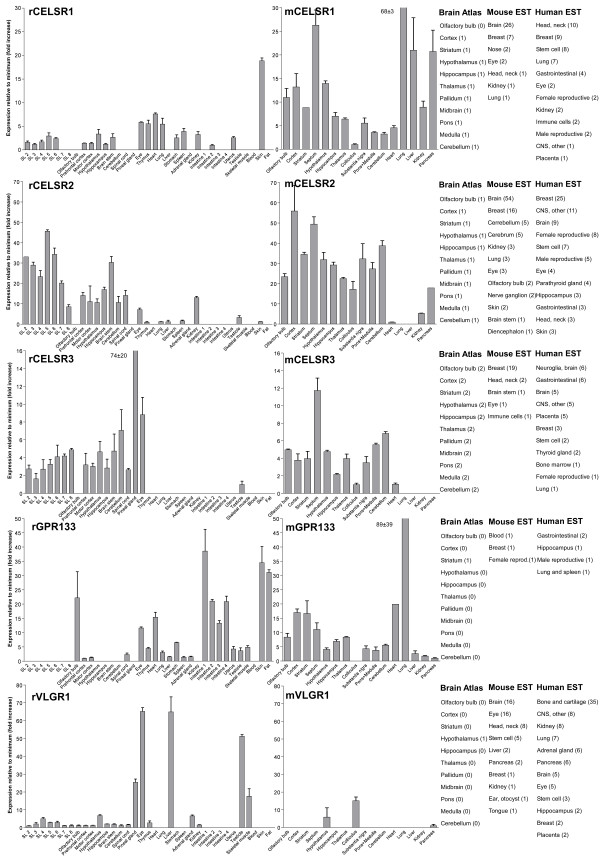
**Expression data of *Adhesion *GPCRs**. Relative expression of rat (r) and mouse (m) *Adhesion *GPCRs: CELSR1, CELSR2, CELSR3, GPR133 and VLGR1 obtained with quantitative real-time PCR. Error bars display standard deviation. The coronal sections of rat brain, marked SL2–SL8, are displayed in Additional File 1. For comparison, in situ hybridization data from mouse (0-no expression, 1-low expression and 2-high expression) [22] as well as mouse and human EST data [23] are displayed on the right side of the figure.

**Figure 7:**
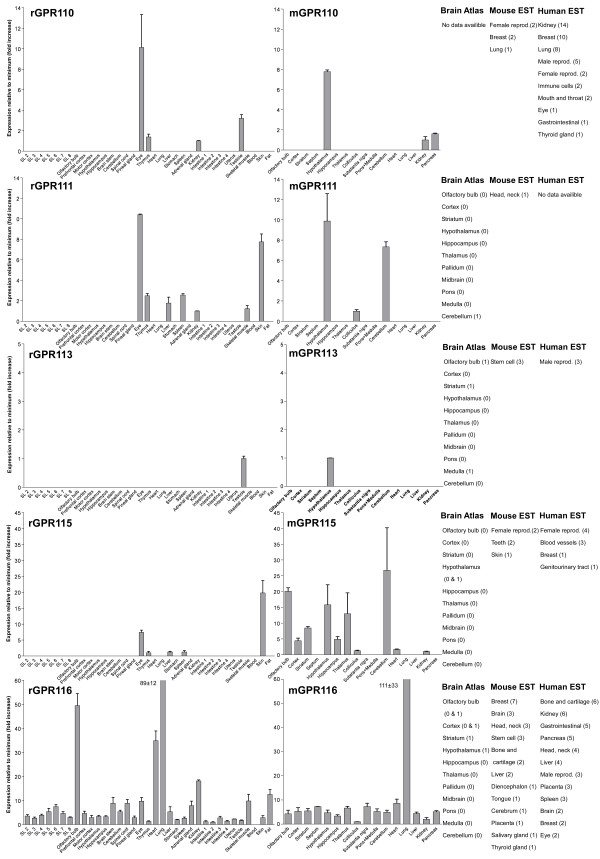
**Expression data of *Adhesion *GPCRs**. Relative expression of rat (r) and mouse (m) *Adhesion *GPCRs: GPR110, GPR111, GPR113, GPR115 and GPR116 obtained with quantitative real-time PCR. Error bars display standard deviation. The coronal sections of rat brain, marked SL2–SL8, are displayed in Additional File 1. For comparison, in situ hybridization data from mouse (0-no expression, 1-low expression and 2-high expression) [22] as well as mouse and human EST data [23] are displayed on the right side of the figure.

We performed an expression analysis of 30 rat and mouse *Adhesion *GPCR family members in a range of central and peripheral tissues. Seven coronal sections of rat brain (Additional file 1), a number of brain regions and peripheral tissues were isolated from mice and rats and used for RNA isolation and cDNA synthesis. The expression values of four housekeeping genes were used to calculate normalization factors for mouse and rat cDNA. Relative expression values displayed as a fold increase from the detected minimum expression for rat and mouse *Adhesion *GPCRs are presented in Figures 2, 3, 4, 5, 6, 7. Expression of BAI1 and BAI2 from group I was detected only in brain regions of rat and mouse. BAI3 transcripts were detected mostly in brain, with very low levels in some peripheral tissues (Figure 2). The expression data of BAI receptors is in agreement with previously published results [25,26]. GPR56, GPR64, GPR126 and VLGR1 from group II were detected in central and peripheral tissues, whereas GPR97, GPR112, GPR114 and GPR128 were detected in peripheral tissues (Figures 2, 3 and 6). The members of group III LEC1, LEC2, LEC3 and ETL were expressed ubiquitously in both rat and mouse, whereas LEC2 and LEC3 had higher levels in CNS compared to levels in the periphery (Figure 4). The members of group III CD97, EMR1 and EMR4 were detected mostly in peripheral tissues and at very low levels in the brain (Figures 4, 5). The expression profile of receptors from *Adhesion *group III is in agreement with previous reports [27-31]. The member of group IV GPR123 displayed central expression, while the other members GPR124 and GPR125 showed ubiquitous expression (Figure 5). The members of group V CELSR2 and CELSR3 were detected mainly in CNS, while CELSR1 was found both in central and peripheral tissues (Figure 6). These results confirmed previously published expression data of CELSR receptors [32,33]. Group VI member GPR133 was detected in both CNS and periphery (Figure 6). GPR144, which is a pseudogene in rat, was not detected in any of the examined mouse tissues (results not shown). Group VII members GPR110, GPR111, GPR113 and GPR115 were detected in only few peripheral tissues (Figure 7). GPR116 was detected in all analyzed tissues, except blood, but mainly at low levels (Figure 7).

## Discussion

In this paper we present for the first time the expression chart of 30 members of *Adhesion *GPCRs in mouse and rat. Together with EST data from human and mouse [23] and previously published Allen Brain Atlas in situ hybridization data [22] this provides the most comprehensive information about the expression pattern of the *Adhesion *family of GPCRs (Figures 1, 2, 3, 4, 5, 6, 7).

*Adhesion *receptors is an evolutionary conserved group, for example an *Adhesion *like genes have been described in fruit fly (*Drosophila melanogaster*) [34] and in the choanoflagellate (*Monosiga brevicollis*) [35]. According to our phylogenetic analysis based on TM regions of *Adhesion *receptors they formed seven groups (Figure 1), which is in agreement with previously published studies [2]. Despite that phylogeny was based only on TM regions the N-termini of the receptors in each group had similar domain types and organization. Surprisingly, our results showed that the members within each group also share considerable similarities in their expression profiles (Figure 1). So according to our results group I with BAI1, BAI2, BAI3; group IV with GPR123, GPR124, GPR125; group V with CELSR1, CELSR2, CELSR3 and even LEC1–3 and ETL1 which form a cluster within group III showed predominant expression in the brain of rat and mouse (Figures 2, 4, 5 and 6).

The *Adhesion *GPCR group containing GPR123, GPR124 and GPR125 is one of the least studied groups. The detailed CNS profile of GPR123 was recently published by Lagerström et al. with some indications of important neuronal functions [18]. It is therefore very exciting that our results display wide expression in the rat and mouse brain for all 3 members of this group. In contrast to GPR123, which displayed specific central expression, GPR124 and GPR125 displayed also very wide peripheral expression (Figure 5). It is very interesting that both the human and mouse ESTs for GPR125 are detected in stem cells and it was recently reported that GPR125 could be used therapeutically as a stem cell marker for generation of vessels [19]. The N-terminals of GPR124 and GPR125 contain GPS, leucine-rich repeats, Ig domain and hormone binding domain in contrast to GPR123, which is lacking all of these domains. It is therefore tempting to speculate about if any of these domains have a role in peripheral actions of GPR124 and GPR125. Other proteins with leucine-rich repeats like LINGO-1 and LRRK2 have been shown to play a role in structural and functional integrity of neurons involved in Parkinson's disease [36]. The members of this group are present in teleosts [18], amphioxus and fruit fly (unpublished data). As this group is present within most species with a nervous system and have a wide CNS expression, members from this family could have their main function within the CNS.

Similarly to the centrally expressed *Adhesion *GPCR genes, the genes expressed predominantly in peripheral tissues are also found in phylogenetic clusters. High levels of GPR128 transcripts were detected in stomach, and all parts of intestine. This is in agreement with the observation that the highest number of ESTs for GPR128 from human and mouse are found in the gastrointestinal (GI) tract (Figure 3). The GPR128 is unlikely to play a role in immune system regulation as it is not detected in high levels in immune system specific organs. The GI specific expression profile of GPR128 could possibly indicate a function in cell migration during development of GI tract endothelium. Two other genes from group II, GPR97 and GPR114, were also expressed in the GI tract but they were also detected at high levels in thymus, spleen and blood (Figure 3). In addition, the highest number of ESTs for these genes was detected in immune cells, spleen and bone marrow. Altogether this indicates that GPR97 and GPR114 most probably are involved in functions of immune system in humans and rodents.

GPR56, GPR64 and GPR126 from *Adhesion *GPCR group II displayed ubiquitous expression (Figures 2, 3). GPR56 is most studied of these receptors. It is reported to play a role in development of human cerebral cortex [10] as well as tumor development [12]. According to our results, the expression of GPR56 is highest in mouse pancreas and rat kidney. GPR64 has been described to regulate the function of the male reproductive system [13]. Our results together with data from Allen Brain Atlas [22] display expression of GPR64 in hypothalamus, brain stem, substantia nigra and spinal cord. This was surprising to us as several publications have described GPR64 as an epididymal specific receptor. Therefore it is likely that GPR64 could also have an additional function in CNS.

Mutations in VLGR1 are known to cause audiogenic seizures in mice [37]. Hence VLGR1 expression in embryonic CNS is widely described not only in mammals but also in other vertebrates like zebrafish [38]. Interestingly, our results indicate that there are common expression sites of VLGR1 in zebrafish embryo and adult rat such as hypothalamus and eye (Figure 6). Such similarity in expression patterns between mammals and teleosts points at remarkable conservation of function of the VLGR1 receptor during vertebrate evolution.

GPR133 and GPR144 are relatively recently discovered [39] and poorly studied *Adhesion *GPCRs. The presence of GPR133 like sequence (XM_001197663) in purple sea urchin (*Strongylocentrotus purpuratus*) indicates a long evolutionary history and an important physiological function. The expression of GPR133 was highest in intestine, skin, adipose tissue and olfactory bulb (Figure 6), which points toward a function in periphery. The inability to detect GPR144 transcripts in mouse tissues verifies that GPR144 is a murine pseudogene.

GPR116 is the only member of the *Adhesion *GPCR group VII with ubiquitous expression (Figure 7). The expression profile of GPR116 with highest transcript levels in lung, olfactory bulb and heart is most similar to the ETL and CD97 receptors. This could be a sign of functional similarities between these receptors. GPR110, GPR111 and GPR115 have common expression site in the rat eye. Two of these receptors, GPR111 and GPR115 also share expression in the rat skin. These receptors have similar structure in their N-termini and similar protein length. It is possible that these receptors play a role in similar peripheral functions like epithelial cell adhesion in eye epithelium and skin.

## Conclusion

It is remarkable that phylogenetic clusters *of Adhesion *GPCRs seem to have either predominant central or peripheral expression in rat and mouse tissues. Though, it is also clear that within the phylogenetic clusters the expression profiles are not identical as *Adhesion *GPCRs are expressed in a large number of tissues. If we analyze the functions of the well studied *Adhesion *GPCRs, it is evident that even if the receptors from the same phylogenetic cluster do play roles in similar physiological processes, they do not have the same function and sometimes can even have opposite functions. One possible explanation for this is that *Adhesion *receptors are very well conserved during evolution and sub-functionalization was a common process among phylogenetic clusters of *Adhesion *GPCRs. *Adhesion *receptors participate in highly complex processes like development of nervous system and immune system response. These processes require often a strict sequence of steps with different types of adhesion molecules participating at the different stages. Altogether the *Adhesion *GPCRs are a unique and fascinating family of membrane receptors with conserved evolutionary history and a number of vital physiological functions.

## Methods

### Animal handling and tissue isolation

A number of adult male and female 129sv mice and Sprague-Dawley rats (Scanbur, BK AB) were kept in separate air-conditioned rooms (12 h dark/light cycle) at 22–23°C in an air humidity of 55%. The animals had free access to water and food pellets (Labfor, Lactamin, Sweden). These conditions were maintained for 7 days, and at the end of the period the animals were sacrificed by decapitation (rats) or cervical dislocation (mice) between 3 and 6 hours into the light period. All animal procedures were approved by the Uppsala Ethics Committee and followed the guidelines of European Communities Council Directive (86/609/EEC). Different brain regions and peripheral tissues were isolated. With regard to the rat brains, they were taken out immediately after decapitation. Two whole rat brains were sliced into coronal cross sections (approximately 3 mm thick) using a rat brain matrix (Pelco International, Canada) as presented in Fig. 1. The remaining rat brains were dissected in more detail. The entire hypothalamus was carefully removed by lifting it from the brain with a slightly curved forceps. Thereafter the following regions were manually dissected with the guidance of a rat brain atlas (Paxinos G., Watson C., 1997. The Rat Brain in Stereotaxic Coordinates. Academic Press, San Diego, CA): prefrontal cortex, motor cortex and hippocampus. The first section (approximately bregma 5.20–4.20 mm) was denoted prefrontal cortex. Motor cortex was collected from sections approximately between bregma 4.20–2.70 mm and included primary and secondary motor cortex. The hippocampus was collected from several sections and therefore comprised the hippocampus CA1–3 and the dentate gyrus. The tissues were immersed into RNA-later solution (Ambion, USA), kept at room temperature for 1 hour and thereafter stored at -80°C until further processed.

### RNA isolation and cDNA synthesis

Individual tissue samples were homogenized by sonication in TRIzol reagent (Invitrogen, Sweden) using a Branson sonifier. Chloroform was added to the homogenate, which was then centrifuged at 10 000 g at 4°C for 15 minutes. The water phase was collected and RNA was precipitated with isopropanol. The pellets were washed with 75% ethanol, air dried and dissolved in RNAse free water. DNA contamination was removed by treatment with DNAse I (Roche Diagnostics, Sweden) at 37°C for 4 h where after the DNAse I was inactivated by heating the samples to 75°C for 15 minutes. Absence of DNA contamination in the RNA was confirmed by PCR and RNA concentration was determined using a Nanodrop^® ^ND-1000 Spectrophotometer (NanoDrop Technologies, Delaware, USA). cDNA was synthesized with M-MLV reverse transcriptase (Invitrogen, Sweden) using random hexamers as primers according to the manufacturers' instructions. The quality of the cDNA was confirmed by PCR.

### Primer design and evaluation

Sequences for rat and mouse house keeping genes and all known *Adhesion *GPCR gene sequences were downloaded from GenBank. GenBank accession numbers are presented in Additional File 2. All primers were designed with Beacon Designer v4.0 (Premier Biosoft, USA) and positioned within TM regions of the *Adhesion *GPCRs. The primers sequences for rat and mouse *Adhesion *GPCRs and house keeping genes are displayed in Additional File 2.

### Quantitative Real-Time PCR

The cDNA was analyzed in quantitative real-time PCR with a MyiQ thermal cycler (Bio-Rad Laboratories, Sweden). Each real-time PCR reaction with a total volume of 20 μl contained cDNA synthesized from 25 ng of total RNA; 0.25 μM of each primer, 20 mM Tris/HCl (pH 8.4), 50 mM KCl, 4 mM MgCl_2_, 0.2 mM dNTP, SYBR Green (1:50 000). Real-time PCR was performed with 0.02 u/μl Taq DNA polymerase (Invitrogen, Sweden) under the following conditions: initial denaturation at 95°C for 4 min, followed by 50 cycles at 95°C for 15 s, 55–62°C for 30 s (optimal annealing temperature) and 72°C for 30 s. This was followed by 84 cycles at 55°C for 10 s (increased by 0.5°C per cycle). All real-time PCR experiments were performed in duplicate. A negative control for each primer pair and a positive control with 25 ng of rat and mouse genomic DNA, respectively, was included on each plate.

### Data analysis and relative expression calculations

MyiQ software v 1.04 (Bio-Rad Laboratories, Sweden) was used to analyse real-time PCR data and derive threshold cycle (Ct) values. Melting curves were analysed to confirm that only one product was amplified and that it was different from the negative control. LinRegPCR [40] was used to calculate PCR efficiencies for each sample. After that Grubbs' test (GraphPad, USA) was applied to exclude the outliers and calculate average PCR efficiency for each primer pair. The delta Ct method [41] was used to transform Ct values into relative quantities with standard deviations and the highest expression was normalized to 1. The GeNorm software [42] was used with results from four housekeeping genes in order to calculate normalization factors for every tissue to compensate for differences in cDNA amount. Thereafter the normalized quantities were calculated and minimum expression was set to 1. All relative expression values were displayed as fold increase from the detected minimum expression.

## Authors' contributions

TH designed the study, analyzed the data and drafted the manuscript. FO and OS performed the real-time PCR on rat, respectively mouse tissues and made the calculations. TE and JA carried out the dissection of mouse tissues. ER and RF performed the dissection of rat tissues. HBS conceived of the study and helped to draft the manuscript. RF participated in the design of the study and helped to draft the manuscript. All authors read and approved the final manuscript.

## Supplementary Material

Additional file 1 Schematic presentation of coronal sections of rat brain. The sections used in this study are marked SL2–SL8. Click here for file

Additional file 2 Primers used for real-time PCR analysis. Table includes gene names for mouse (m) and rat (r) *Adhesion *GPCRs and house-keeping genes (*), GenBank accession numbers and primer sequences. NA – not available. Click here for file
