# Supplementary figures and images for: Expression profile of the entire family of Adhesion G protein-coupled receptors in mouse and rat
Source: BMC Neurosci. 2008 Apr 29;9:43. doi: 10.1186/1471-2202-9-43 (PMC2386866; doi:10.1186/1471-2202-9-43)

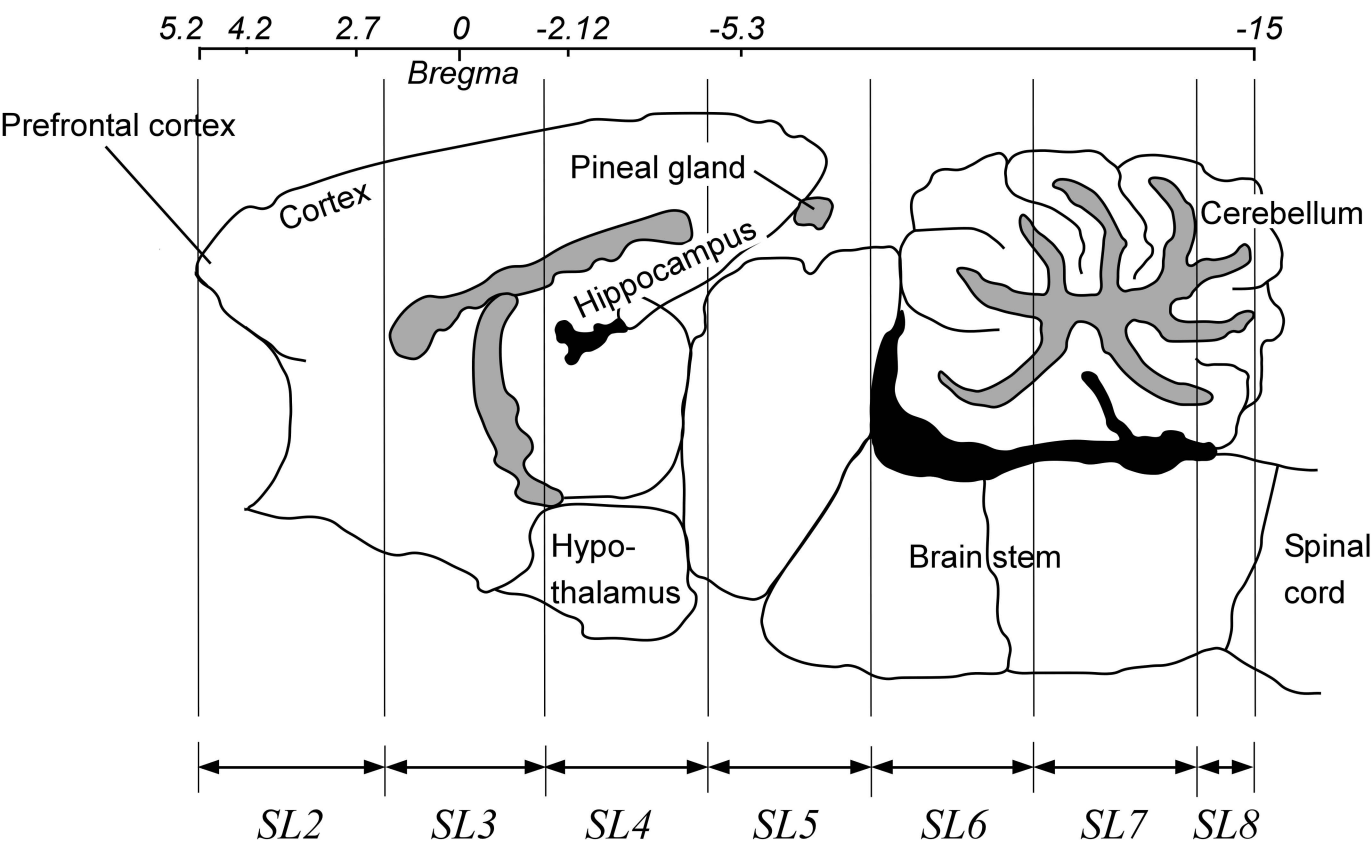

Supplement: Additional file 1 — Schematic presentation of coronal sections of rat brain. The sections used in this study are marked SL2–SL8. [file 1471-2202-9-43-S1.pdf]
