# Supplementary material for: Expression profile of the entire family of Adhesion G protein-coupled receptors in mouse and rat
Source: BMC Neurosci. 2008 Apr 29;9:43. doi: 10.1186/1471-2202-9-43 (PMC2386866; doi:10.1186/1471-2202-9-43)
Supplement: Additional file 2 — Primers used for real-time PCR analysis. Table includes gene names for mouse (m) and rat (r) Adhesion GPCRs and house-keeping genes (*), GenBank accession numbers and primer sequences. NA – not available. [file 1471-2202-9-43-S2.pdf]

Name	Accesion No.	5’	3’
mBAI1	NM_174991.3	tggatgtctgctgtgcttgctg	acgatgacgaagccctccagtg
rBAI1	XM_343260	tggatgtctgctgtgcttgctg	acgatgacgaagccctccagtg
mBAI2	NM_173071.1	tcctgcgtggtactgcctctcc	gcacggcggtgatgacaaagc
rBAI2	XM_232778	tcctgcgtggtactgcctctcc	gcacggcggtgatgacaaagc
mBAI3	NM_175642.3	gacagataaacgctccatattg	acctctctccgaagaatgc
rBAI3	XM_217367	gacagataaacgctccatattg	acctctctccgaagaatgc
mCD97	NM_011925.1	cctgatctgcctgctgctgtg	cttcattctccacgccgaccag
rCD97	NM_001012164.1	ctgcctgctgctctgtatcc	cccttcattctcaacaccaacc
mCELSR1	NM_009886.2	atgccgccctgtccttgtc	gaggttcttgtggatgctgtgc
rCELSR1	XM_001078424	tggtgtccttcgtcctgctctc	agccgcaatgaggttcttgtgg
mCELSR2	NM_017392.3	agtcatcgctatcctgctg	cgcatgggactggcattg
rCELSR2	XM_001070611	gcacttcctgtacctttg	agcatgtagtagaaacgc
mCELSR3	NM_080437.2	gctcttttctgctccttctgc	cagcatggaggtagtggaag
rCELSR3	NM_031320.1	gctcttttctgctccttctgc	cagcatggaggtagtggaag
mEMR1	NM_010130.1	ccttgcctgcttcttctggatg	agcatcttgatgttgcgagagc
rEMR1	NM_001007557.1	ccttgcctgcttcttctggatg	agcatcttgatgttgcgagagc
mEMR4	NM_139138.1	gctctccatctgccttttcctg	tagcccttccagaaacatccac
rEMR4	NM_001007558.1	tgtgggactcagtctttctctc	tggagtgtcgtgctggtattc
mETL	NM_133222.1	agcaccaggaccacgattcac	gccagcaatgatagagcagacc
rETL	NM_022294.1	ttgggctcatctataacaaggg	gccgagaatccaacaactacag
mGPR110	NM_133776.1	gcagaccaagagaagccaaacc	cagccacacagactccagaagg
rGPR110	NA	catacatagggctgggcgtctc	ttgcgtgtgtaggaggtttggc
mGPR111	NM_001033493.1	tgttcacttcttttacctgtcc	acgacttgggcagtgtatg
rGPR111	NA	ccctgcccaagtcgtgtctg	agcaagtgtgatgacagcgatg
mGPR112	NM_001033327.1	tagcctttctggtgaactcctg	ccatccaagtcaaagacacgag
rGPR112	NA	gccttaccaccagagtctttgc	gcactgaatggactgtgaatgg
mGPR113	NM_001014394.2	aggggaaatgctttctgaacgg	cacagcgatgacaaggaccag
rGPR113	NA	gcgggaggggaaatgcttgc	gcacagcaatgacaaggaccag
mGPR114	NM_001033468.1	tcgggcacgggagaaggc	tgaggcagcaggaacacacc
rGPR114	NA	ccacagtgcccaggtcagtc	cgacgcccatccaggtaagg
mGPR115	BC089564	tcatcggctccaacttcagtgc	agcagggctttgaagagcatcc
rGPR115	NA	tcatcggctccaacttcagtgc	agcagggctttgaagagcatcc
mGPR116	NM_001081178.1	tgaggcagcaggaacacacc	tgtttcgttgagtgggtagcg
rGPR116	NM_139110.1	ccaccaaagatgtcactgttcac	gcacagaccactggcttcc
mGPR123	BC056493	tctccttgcctctgtcatcacc	cggcgtggaagcagaagttg
rGPR123	NA	ggcagtgttggtggtcatg	ccgttggcatcaagtgtgg
mGPR124	NM_054044.1	ctcttccttgtcgtcactg	cagcaatcaagtagaaccg
rGPR124	NA	ggaggtaggagtgtcaggag	cagtggtacagcggaatcg
mGPR125	BC052391	atgcttgtgaacctgtgctttc	cgctggcatttctggtctgg
rGPR125	NA	agggaggacgtgaggctttc	aggtgggaggctggacattg
mGPR126	NM_001002268.1	ggaatggaaagagaagcaaccg	cccaggcaaagaaagcaaacc
rGPR126	NA	attctgctggattcaggatc	ctgtaccatgaccacgatg
mGPR128	NM_172825.1	accattgaaaccccgaatccc	agagttgtgtagcactgagtcc
rGPR128	NA	accagaaacagacaccattgaaac	tgtggcactgagtccattcc
mGPR133	NM_001081342	gtcatctcccacatcagcactg	ggtcactgacggcaagcac
rGPR133	NA	catagggtgtgagcacgactg	cttgatctcgtagggagtcagc
mGPR144	NA	cactctacacccagactctacc	tcctcagcagcgactcctc
mGPR56	NM_018882.2	tctgctctggcttgtgtcttc	aggttcatgtggactttgatgg
rGPR56	NM_152242.1	gcacatgaacctgcttctgg	ggtgcgacaggctgcttc
mGPR64	BC116644	cctgcttctgctcaacctgatc	agaaatacagccacggcaatgc
rGPR64	NM_181366.1	gcttctgctcaacctggtcttc	agaaatacagccacggagatgc
mGPR97	NM_173036.2	cctgtttctcctgaatcttacc	gaagacacagagcagaaagtag
rGPR97	NA	cctcttcggtgctgtggtgctg	ccctgtcctttgcccgctgtg
mLEC1	NM_001081298.1	atcgttgtctccctcgtctgc	agtattgcggtcactttgtagcc
rLEC1	NM_134408.1	tggatgtgcctagaaggtgtgc	cactgtggcagggaagaggtac
mLEC2	NM_181039.1	tcgtttgtcattgtggtgaacc	aggcgactagagtcaggcttg
rLEC2	NM_022962.1	aacaaggagtcagtagtaatgg	cgcagtgaaagacaaagatg
mLEC3	NM_198702.2	ttgctgtcccttgtctgtctcc	aatggtgttgcggtcactctgg
rLEC3	NM_130822.1	cgtgggtcggaatcctgttgtc	aatggtgttacggtcgctctgg
mVLGR1	NM_054053.2	tttcgtggtgattctcctc	accatgaataagtccatagatc
rVLGR1	NA	gctgctcttgtgcctctaatg	tcatcgtaacctttccactgtg
mACTIN, beta*	NM_007393.1	ccttcttgggtatggaatcctgtg	cagcactgtgttggcatagagg
rACTIN, beta*	NM_031144	cactgccgcactctcttcct	aaccgctcattgccgatagtg
rCYCLOPHILIN*	NM_008907.1	gagcgttttgggtccaggaat	aatgcccgcaagtcaaagaaa
mGAPDH*	NM_008084.2	gccttccgtgttcctacc	gcctgcttcaccaccttc
mRPL19*	NM_009078.1	aatcgccaatgccaactc	ggaatggacagtcacagg
rRPL19 *	NM_031103	tcgccaatgccaactctcgtc	agcccgggaatggacagtcac
mTUBULIN, beta*	NM_011655.3	agtgctcctcttctacag	tatctccgtgttaagtgc
rTUBULIN, beta*	NM_173102	cggaaggaggcggagagc	agggtgcccatgccagagc
